# Sex‐stratified genome‐wide meta‐analysis identifies novel loci for cognitive decline in older adults

**DOI:** 10.1002/alz.088281

**Published:** 2025-01-03

**Authors:** Vibha Acharya, Kang‐Hsien Fan, Beth E. Snitz, Mary Ganguli, Steven T. DeKosky, Eleanor Feingold, M. Ilyas Kamboh

**Affiliations:** ^1^ School of Public Health, University of Pittsburgh, Pittsburgh, PA USA; ^2^ School of Medicine, University of Pittsburgh, Pittsburgh, PA USA; ^3^ University of Pittsburgh School of Public Health, Pittsburgh, PA USA; ^4^ College of Medicine, University of Florida, Gainesville, FL USA; ^5^ McKnight Brain Institute, University of Florida, Gainesville, FL USA

## Abstract

**Background:**

Many complex traits and diseases show sex‐specific biases in clinical presentation and prevalence. For instance, two‐thirds of AD cases are female. Studies suggest that women might have higher cognitive reserve but steeper cognitive decline in older age. Cognitive traits have substantial genetic influence. Cognitive impairment could vary among sexes due to sex‐specific genetic factors. To understand the sex‐specific genetic architecture of cognitive decline, we performed genome‐wide association study (GWAS) on sex‐stratified data and assessed a SNP‐sex interaction.

**Method:**

We derived inter‐individual specific slopes using linear mixed effect model adjusting for age, education, and sex across five cognitive domains (attention, memory, executive function, language, visuospatial function) and global cognitive function in 3021 older adults aged ≥65 years (females = 1545, males = 1476) belonging to three prospective cohorts: Gingko Evaluation of Memory (GEM), Monongahela‐Youghiogheny Heathy Aging Team (MYHAT), and Monongahela Valley Independent Elders Survey (MoVIES). GWAS was conducted on each cognitive phenotype in each cohort adjusting for the first four genetic principal components. Gene‐based analysis was also conducted to discover genes specific to biological sexes.

**Result:**

We identified a novel genome‐wide significant (GWS) intergenic locus for decline of memory in males near *RPS23P3* on chromosome 4 (top SNP: rs6851574; MAF = 0.39; P_male_ = 4.10E‐08, b_male_ = ‐0.19; P_female_ = 6.13E‐01, b_female_ = ‐0.017; P_interaction_ = 3.76E‐04). This intergenic region has previously been associated with other cognitive related traits such as educational attainment, anatomical brain aging and brain shape. We also identified a subthreshold GWS locus for decline of executive function in females near *NDUFA12* on chromosome 12 (top SNP: rs11107823; MAF = 0.12; P_female_ = 9.35E‐08, b_female_ = 0.28; P_male =_ 0.151, b_male_ = ‐0.077; P_interaction_ = 7.42E‐06). *NDUFA12*/rs11107823 is cis‐eQTL for *NDUFA12*, *NTN4* and *VETZ* in blood and brain tissues. *NDUFA12* codes for a gene involved in oxidative phosphorylation in mitochondria and was also found to be suggestively associated with decline of executive function in gene‐based analysis in females (P = 1.40E‐04).

**Conclusion:**

In this sex‐stratified analysis of cognitive decline, we identified two novel loci affecting cognition. Our study illustrates that sex‐aware genetic studies can help in the identification of novel genetic loci and enhance sex‐specific understanding of cognitive aging.

